# Supplementary material for: An In Vitro Model to Assess Early Immune Markers Following Co-Exposure of Epithelial Cells to Carbon Black (Nano)Particles in the Presence of S. aureus: A Role for Stressed Cells in Toxicological Testing
Source: Biomedicines. 2024 Jan 8;12(1):128. doi: 10.3390/biomedicines12010128 (PMC10813740; doi:10.3390/biomedicines12010128)
Supplement: Supplementary file 1 [file biomedicines-12-00128-s001.zip › biomedicines-2764834-supplementary.pdf]

Supplementary Figure legends

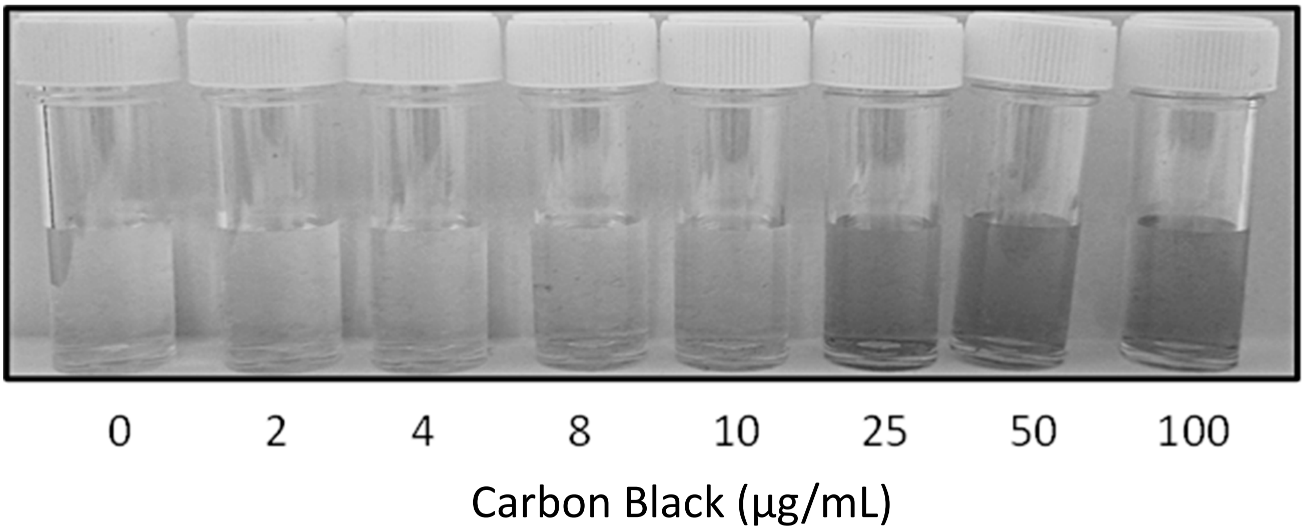

Supplementary Figure S1: Visual appearance of Carbon Black suspensions
Carbon Black (AROSPERSE® 15) at final concentrations of 2, 4, 10, 25, 50 and 100µg/mL were suspended in water, decanted to a 5 mL bijou, vortexed and macroscopically imaged.

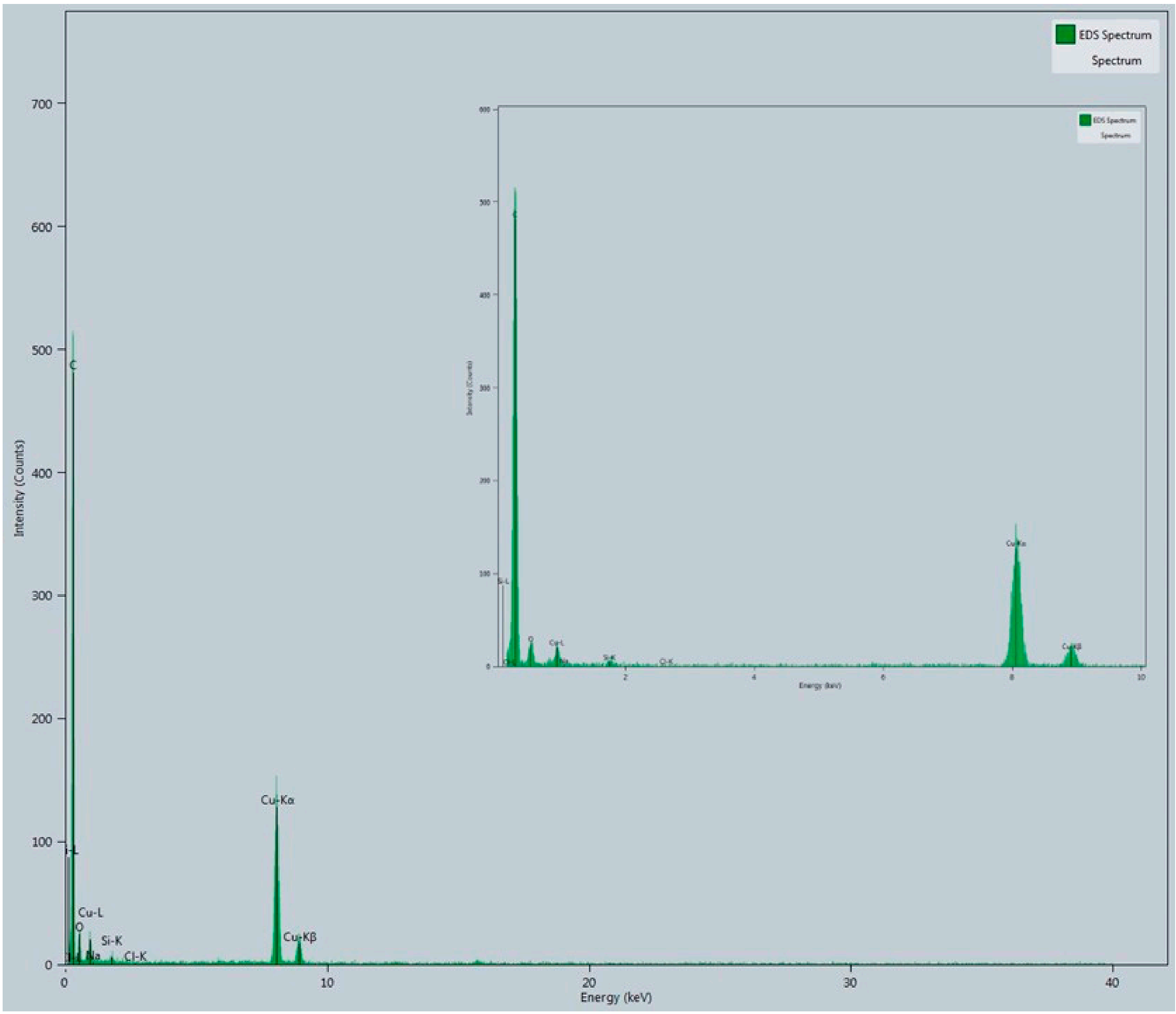

Supplementary Figure S2: Energy Dispersive X-ray (EDX) spectroscopy of Carbon Black

Determination of the chemical composition of Carbon Black (AROSPERSE® 15) particles indicated by energy-dispersive X-ray analysis. Analysis of CB confirmed the presence of elemental carbon in high quantities. Copper peaks relate to the composition of the TEM drop cast mounting grid apparatus. Main spectrum: Wide sweep (0-40keV) X-ray spectrum of energy released from CB surface during TEM analysis. Inset: Discrete sweep (0-10KeV) magnification of 'carbon' specific energy release.

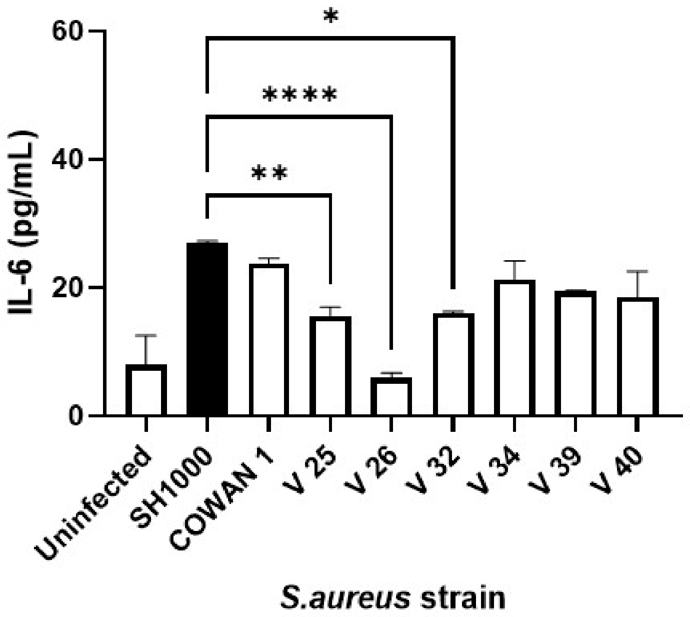

Supplementary Figure S3: Selection of IL-6-inducing *S. aureus* strain

Human HaCaT skin epithelial cells were stimulated with eight strains of *S. aureus* for six hours. Supernatants were collected and the concentration of IL-6 determined by ELISA. Results are expressed as the mean ± SEM of 4 experiments (n=4). Differences between treatments were calculated using an ANOVA multiple comparison test with a Tukey's post hoc test with *p<0.05, **p<0.01, and ****p<0.0001 levels considered significantly different. Black bar shows *S. aureus* SH1000 response.

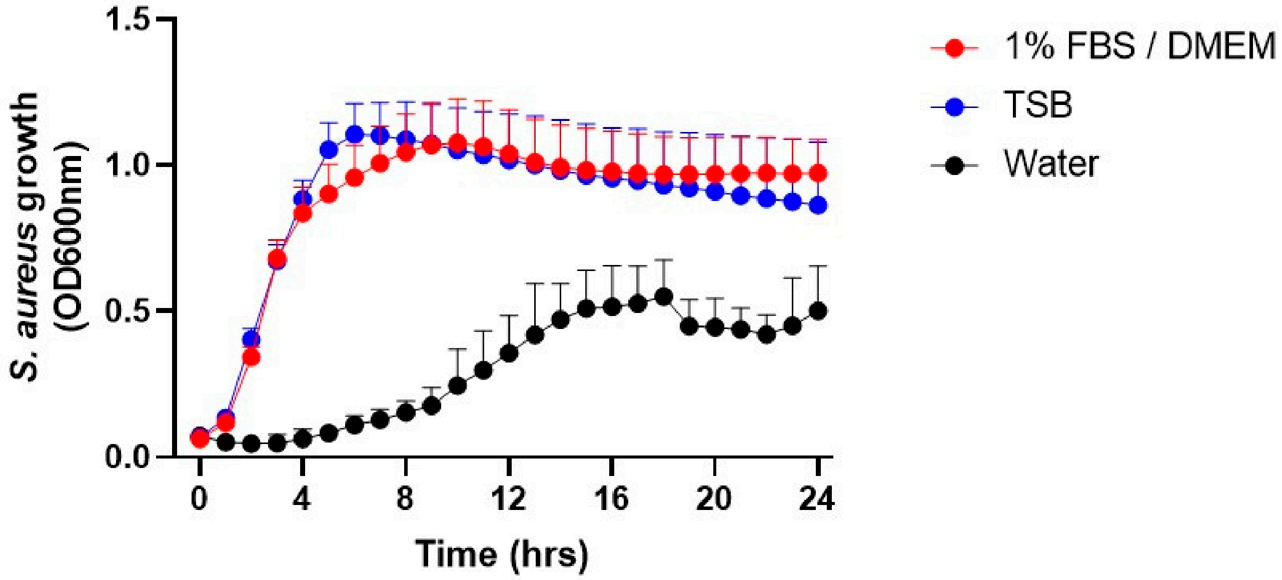

Supplementary Figure S4: The effect of media on the growth of *S. aureus*, SH1000.
One colony of *S. aureus*, SH1000 was inoculated into 5mL of TSB and grown overnight at 37°C. Then, a 1:100 dilution of this preculture was added to water, TSB and 1% FBS/DMEM and the optical density (600nm) was measured over the next 24 hours to assess growth. Results are expressed as Mean ± SEM of 3 experiments (n=3). Differences between treatments were calculated using the Kruskal Wallis multiple comparison test. A $p<0.05$ between two means was considered significantly different.

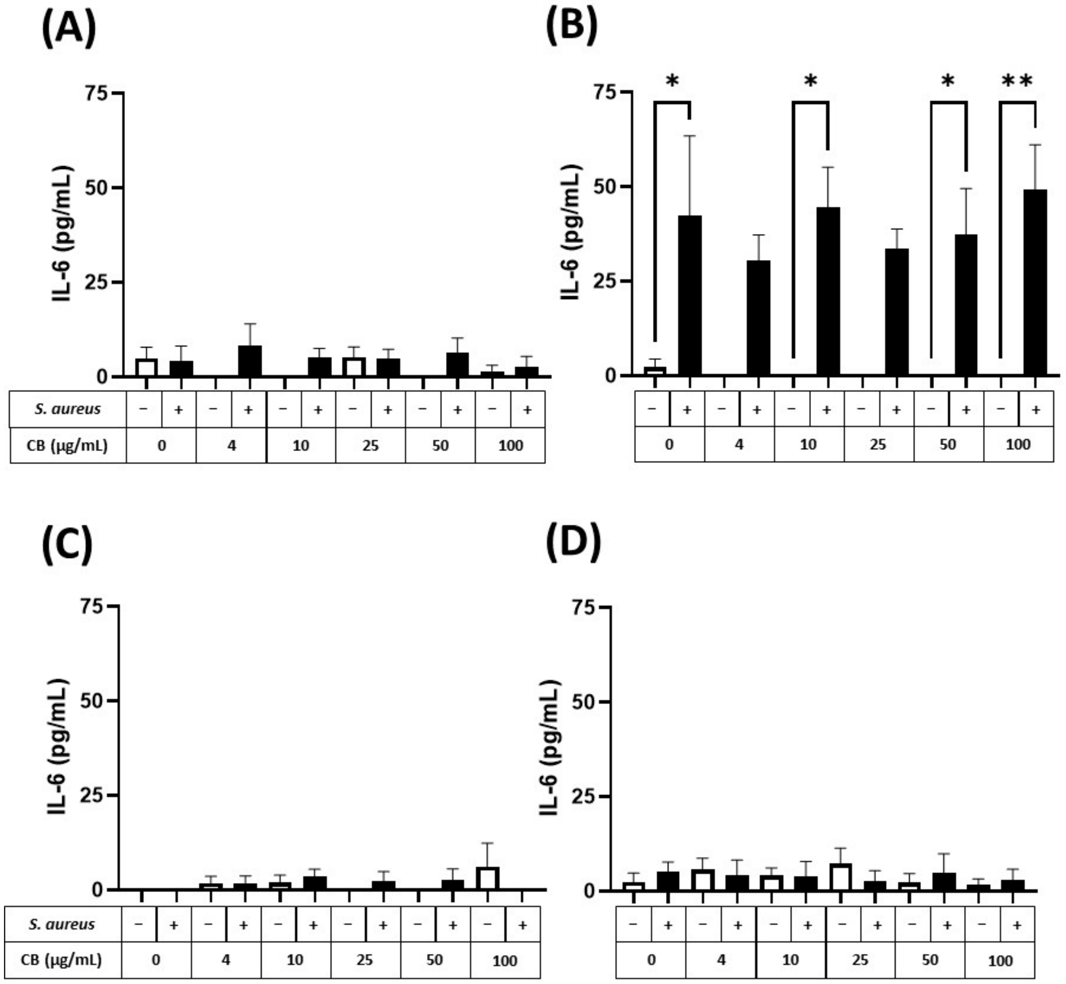

Supplementary Figure S5: The effect of Carbon Black and *S. aureus* SH1000 on IL-6 production in epithelial cells

Human HaCaT skin (A and B) and A549 lung (C and D) epithelial cells were stimulated with increasing concentrations of Carbon Black (0, 4, 10, 25, 50 and 100µg/mL) in combination with *S. aureus* for 5 (A and C) and 24 (B and D) hours. Supernatants were collected and the concentration of IL-6 determined by ELISA. Results are expressed as the mean ± SEM of 4 experiments (n=4). Differences between treatments were calculated using an ANOVA multiple comparison test with a Tukey's post hoc test with *p<0.05, and **p<0.01 levels considered significantly different. Black and white bars represent treatments with and without *S. aureus* SH1000 respectively.

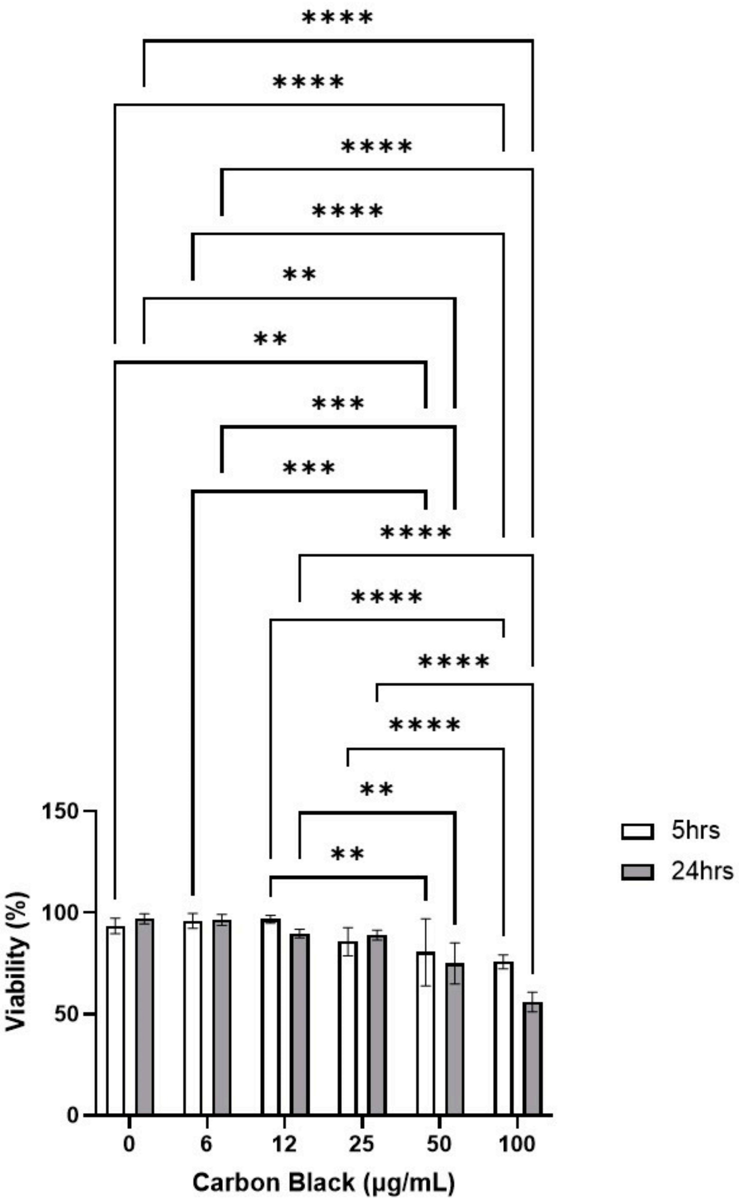

Supplementary Figure S6: The effect of Carbon Black on HaCaT epithelial cell viability
Human HaCaT skin epithelial cells were stimulated with increasing concentrations of Carbon Black (0, 4, 10, 25, 50 and 100µg/mL) for 5 (light bars) and 24 (dark bars) hours. Cells were gently removed by trypsinisation, and viability assessed through trypan blue staining and countess™ automatic cell counting. Results are expressed as the mean ± SEM of 4 experiments (n=4). Differences between treatments were calculated using an ANOVA multiple comparison test with a Tukey's post hoc test with *p<0.05, **p<0.01, ***p<0.001 ****p<0.0001 levels considered significantly different.

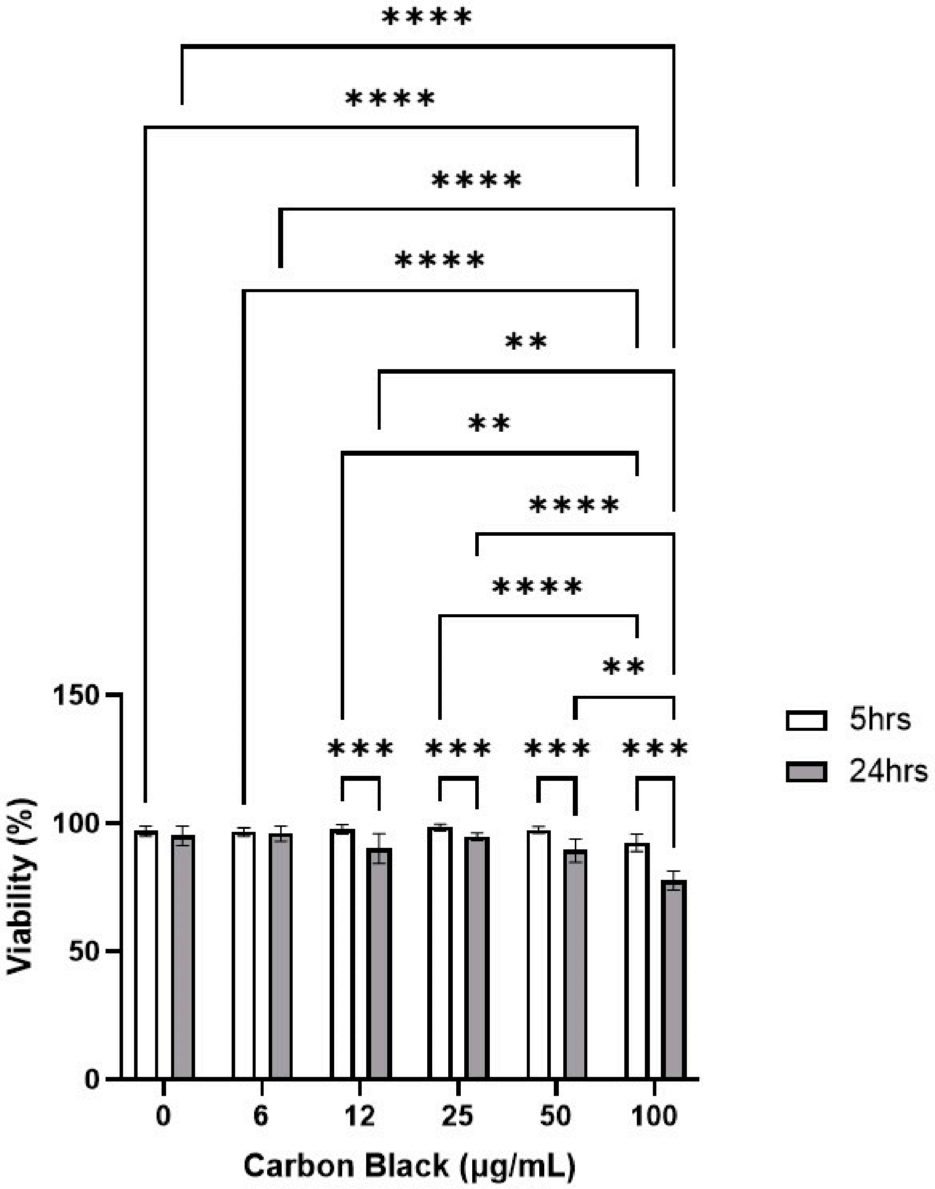

Supplementary Figure S7: The effect of Carbon Black on A549 epithelial cell viability
A549 lung epithelial cells were stimulated with increasing concentrations of Carbon Black (0, 4, 10, 25, 50 and 100µg/mL) for 5 (light bars) and 24 (dark bars) hours. Cells were gently removed by trypsinisation, and viability assessed through trypan blue staining and countess™ automatic cell counting. Results are expressed as the mean ± SEM of 4 experiments (n=4). Differences between treatments were calculated using an ANOVA multiple comparison test with a Tukey's post hoc test with *p<0.05, **p<0.01, ***p<0.001 ****p<0.0001 levels considered significantly different.

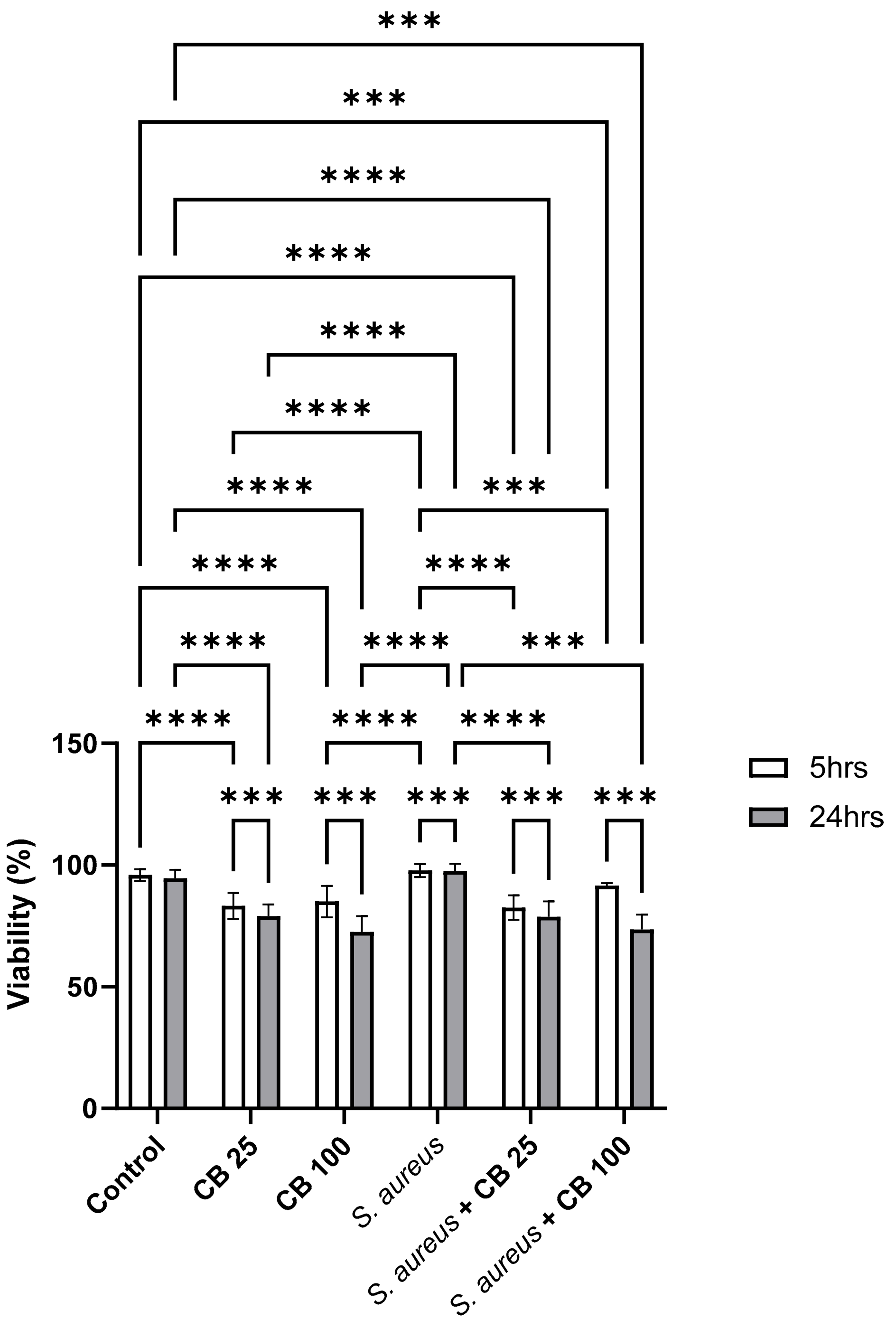

Supplementary Figure S8: The effect of Carbon Black and *S. aureus* SH1000 on HaCaT epithelial cell viability

Human HaCaT skin epithelial cells were stimulated with Carbon Black (25 and 100µg/mL) alone or in combination with *S. aureus* for 5 (light bars) and 24 (dark bars) hours. Cells were gently removed by trypsinisation, and viability assessed through trypan blue staining and countess™ automatic cell counting. Results are expressed as the mean ± SEM of 4 experiments (n=4). Differences between treatments were calculated using an ANOVA multiple comparison test with a Tukey's post hoc test with *p<0.05, **p<0.01, ***p<0.001 ****p<0.0001 levels considered significantly different.

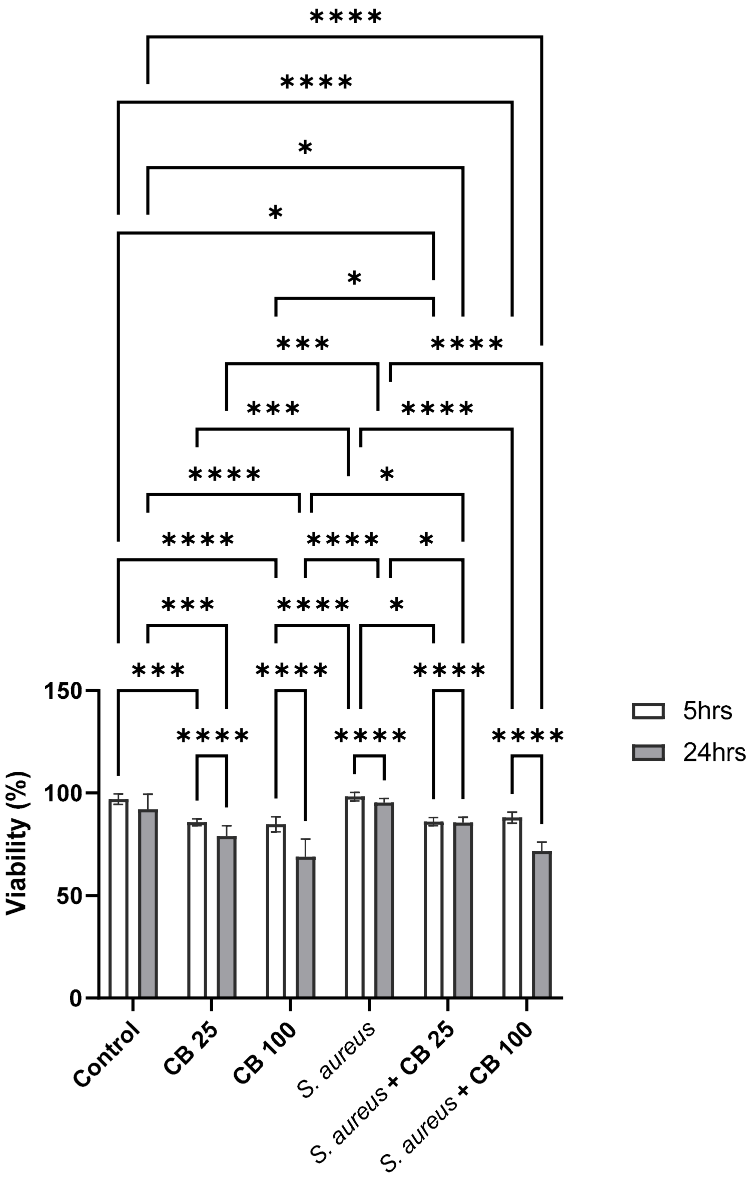

Supplementary Figure S9: The effect of Carbon Black and *S. aureus* SH1000 on A549 epithelial cell viability

Human A549 lung epithelial cells were stimulated with Carbon Black (25 and 100µg/mL) alone or in combination with *S. aureus* for 5 (light bars) and 24 (dark bars) hours. Cells were gently removed by trypsinisation, and viability assessed through trypan blue staining and countess™ automatic cell counting. Results are expressed as the mean ± SEM of 4 experiments (n=4). Differences between treatments were calculated using an ANOVA multiple comparison test with a Tukey's post hoc test with *$p<0.05$, **$p<0.01$, ***$p<0.001$ ****$p<0.0001$ levels considered significantly different.

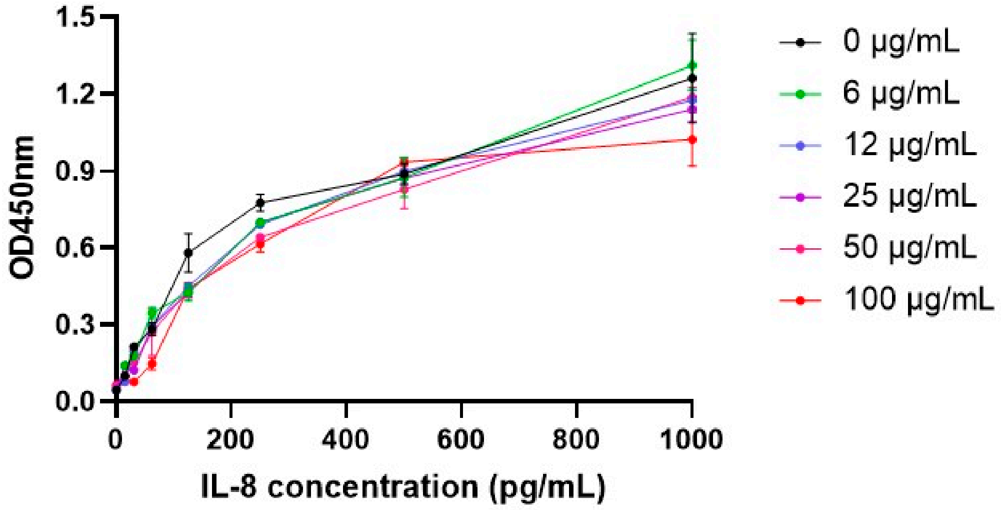

Supplementary Figure S10: Interference of carbon black with IL-8 ELISA
The IL-8 ELISA was carried out according to the outlined methods. To assess for carbon black interference IL-8 standards (0-1000pg/mL) were incubated with increasing doses of CB (0-100µg/mL) and incubated for 24 hours. These incubation mixes were then added to the ELISA as normal samples and the protocol was carried out as normal. Experiments were repeated twice with two duplicates included in each experiment. Differences between treatments were calculated using a two-way ANOVA multiple comparison test with a Kruskal Wallis post hoc test. No significant differences were detected.
